# The Role of Haptic Expectations in Reaching to Grasp: From Pantomime to Natural Grasps and Back Again

**DOI:** 10.3389/fpsyg.2020.588428

**Published:** 2020-12-17

**Authors:** Robert L. Whitwell, Nathan J. Katz, Melvyn A. Goodale, James T. Enns

**Affiliations:** ^1^Department of Psychology, The University of British Columbia, Vancouver, BC, Canada; ^2^Department of Psychology, Brain and Mind Institute, The University of Western Ontario, London, ON, Canada

**Keywords:** grasping, pantomime, haptic feedback, expectations, cognitive supervision

## Abstract

When we reach to pick up an object, our actions are effortlessly informed by the object’s spatial information, the position of our limbs, stored knowledge of the object’s material properties, and what we want to do with the object. A substantial body of evidence suggests that grasps are under the control of “automatic, unconscious” sensorimotor modules housed in the “dorsal stream” of the posterior parietal cortex. Visual online feedback has a strong effect on the hand’s in-flight grasp aperture. Previous work of ours exploited this effect to show that grasps are refractory to cued expectations for visual feedback. Nonetheless, when we reach out to pretend to grasp an object (pantomime grasp), our actions are performed with greater cognitive effort and they engage structures outside of the dorsal stream, including the ventral stream. Here we ask whether our previous finding would extend to cued expectations for haptic feedback. Our method involved a mirror apparatus that allowed participants to see a “virtual” target cylinder as a reflection in the mirror at the start of all trials. On “haptic feedback” trials, participants reached behind the mirror to grasp a size-matched cylinder, spatially coincident with the virtual one. On “no-haptic feedback” trials, participants reached behind the mirror and grasped into “thin air” because no cylinder was present. To manipulate haptic expectation, we organized the haptic conditions into blocked, alternating, and randomized schedules with and without verbal cues about the availability of haptic feedback. Replicating earlier work, we found the strongest haptic effects with the blocked schedules and the weakest effects in the randomized uncued schedule. Crucially, the haptic effects in the cued randomized schedule was intermediate. An analysis of the influence of the upcoming and immediately preceding haptic feedback condition in the cued and uncued random schedules showed that cuing the upcoming haptic condition shifted the haptic influence on grip aperture from the immediately preceding trial to the upcoming trial. These findings indicate that, unlike cues to the availability of visual feedback, participants take advantage of cues to the availability of haptic feedback, flexibly engaging pantomime, and natural modes of grasping to optimize the movement.

## Introduction

Goal-directed grasping is multisensory and integrative in nature. The muscle extensions and contractions that are engaged when we reach out to pick a goal object are specified by motor cortex and rely on computations performed on real-time visual and somatosensory information, stored information about the object’s function, and the agent’s intention (Creem and Proffitt, 2001; Rosenbaum et al., 2001; Frey, 2008; Camponogara and Volcic, 2019; Isa, 2019; Parikh et al., 2020). Studies of the way in which the hand configures while in-flight before the fingers make contact with the object show the hand’s aperture and the orientation of the wrist are smoothly tailored to suit the desired contact posture of the hand (grasp kinematics). Two counter-intuitive findings from studies of grasp kinematics in humans are (1) that the visual routes into and through the posterior parietal cortex (PPC) play a causal role in the planning and execution of visually guided actions (e.g., Tunik et al., 2005; Rice et al., 2007), and (2) grasps can be reliably performed without visual awareness of crucial spatial features of the target such as its location, shape, and size (for review, see Rizzolatti and Matelli, 2003; Jeannerod and Jacobb, 2005; Milner and Goodale, 2006; Frey, 2007; Kravitz et al., 2011).

### The Visual Control of Natural Reaching and Grasping Is Largely Encapsulated From Visual Awareness

Some of the most compelling evidence for this claim comes from individuals with deficits in visual shape perception (e.g., visual form agnosic patients DF, Goodale et al., 1991, 1994b; Karnath et al., 2009) and individuals with cortically blind areas of their visual field due to damage to V1 (e.g., hemianopic patients PGJ, Perenin and Rossetti, 1996; Whitwell et al., 2011) or with damage encompassing the occipital cortex (Striemer et al., 2019; Whitwell et al., 2020). As their clinically defined deficits would predict, these patients perform at chance when reporting object shape, orientation, and/or size. Yet paradoxically, when they reach out to pick up the objects, the way the hand configures while it moves toward the target reliably expresses the target’s spatial properties well-before the hand makes contact with the target (for a comprehensive review of “action blindsight,” see Danckert and Rossetti, 2005). Equally as compelling are findings from cases in which bilateral damage to dorsal stream structures in the PPC leads to deficits in reaching and grasping, despite retention of the ability to make discriminative judgments about the target’s location, size and shape (e.g., Jakobson et al., 1991; Goodale et al., 1994b; Jeannerod et al., 1994).

In normally sighted individuals, grasping resists a number of effects that influence perceptual judgments: grasp aperture resists the target size-distorting influence of illusory backgrounds (e.g, Whitwell et al., 2016, 2018; Carther-Krone et al., 2020; Navon and Ganel, 2020; Smeets et al., 2020; but see Kopiske et al., 2016); it resists Weber’s Law, failing to show a positive relationship between precision and stimulus size (e.g., Ganel et al., 2008; Holmes et al., 2011; Heath et al., 2012; Ozana and Ganel, 2018b; but see Foster and Franz, 2013); and grasp preparation time is not prolonged in the filtering condition of the Garner interference paradigm, in which choice-response times increase when both the relevant and irrelevant dimensions of the target are varied (e.g., Ganel and Goodale, 2003, 2014; Eloka et al., 2015; Freud and Ganel, 2015; Ozana and Ganel, 2018a; but see Löhr-Limpens et al., 2020).

### The Dorsal Stream Is Insufficient for Planning Grasps “for Use”

Much of the early neuropsychological work employed basic, canonical objects such as rectangular blocks, cylinders, and smooth pebble-like shapes. The objects possess few associated semantic features and there was often no additional manual manipulation required. Grasping objects with multiple components, such as tools, poses additional problems, because one must also choose where to direct the hand and how to configure it in a way suitable for the tool’s intended use (Frey, 2007). Thus, the appropriate selection of which subcomponent of the goal object to grasp incorporates semantic and functional information. Functional MRI studies of grasping and using real 3D tools in normally sighted individuals show activity across a wide range of regions that overlap with the praxis representation network, which includes lateral occipital cortex (LOC), the posterior middle temporal gyrus, and supramarginal gyrus of the inferior parietal cortex, in addition to traditional dorsal stream structures in and around the intraparietal sulcus, and premotor cortex (Hermsdörfer et al., 2007; Brandi et al., 2014; Przybylski and Kroliczak, 2017; Styrkowiec et al., 2019). For technical reasons, functional MRI studies of passively observing tools and/or imagining using them are more common, but they often reveal a similar suite of cortical areas in the ventral and dorsal stream, inferior parietal cortex, and frontal and prefrontal areas (MacDonald and Culham, 2015; Chen et al., 2017).

Interestingly, neuropsychological work with DF, who has visual form agnosia following bilateral damage to the object-sensitive LOC in the ventral stream, but a functionally intact dorsal stream (James et al., 2003), has shown that when she reaches out to pick up and demonstrate the use of tools she cannot recognize, her initial grip posture is often inappropriate for using the object, particularly when the functional-end of the tool is oriented toward her. Only after a brief period of haptic exploration does she adjust her grip to better suit her subsequent demonstration of the tool’s use (Carey et al., 1996). DF is similarly impaired when grasping a 3D cross, selecting grasp points across the cross’s intersection rather than opposing ends of one (or the other) of the cross’s composite bars, regardless of the orientation of the cross (Carey et al., 1996). Integrative visual agnosic patient HJA, whose lesions spared early visual cortex but are restricted to the ventral occipito-temporal cortex and extending about halfway into medial temporal cortex, selects inappropriate parts of tools in order to demonstrate their use, much like DF (Humphreys and Riddoch, 2013). Activity in LOC and other ventral stream structures is not only associated with functional or semantic object information, as these structures are also associated with signaling object weight in neurotypical individuals planning grasp-to-lift movements (Gallivan et al., 2014). Taken together, these studies support a role for the ventral stream and other visual areas outside the dorsal stream to assist with the extraction of hidden properties, such as weight, semantic properties and postural schemas associated with a goal-object’s use. They also help to highlight the integrative, multimodal nature of grasping and manipulating objects.

### Is the Dorsal Stream Insufficient for Making Natural-Looking Pantomimed Grasps?

In an early effort to define the limits of the automatic visuomotor modules of the PPC, Goodale et al. (1994a) compared the kinematics of natural and pantomimed grasping in both normally sighted controls and in DF. In controls, pantomime grasps led to longer movement planning times, slower reaches, a narrowing of the grasp aperture, inflation of the aperture’s tendency to increase with target size, and a susceptibility to the effects of target magnitude on the precision of peak grip aperture (PGA) (e.g., Bingham et al., 2007; Fukui and Inui, 2013; Whitwell et al., 2015a). As for DF, Goodale et al. (1994a) reasoned that if pantomime grasps depend on visual awareness of a target object’s 3D properties, then her pantomimed grasp aperture should not scale with the size of the target. In accordance with this prediction, when DF based her pantomime grasps on a short-term memory of a visual preview of the object, her in-flight grip aperture did not covary with the size of the remembered targets (Goodale et al., 1994a; Whitwell et al., 2015a). Nevertheless, when DF was instructed to direct her pantomimed grasps beside a nearby visible target, her grasp aperture covaried reliably with the target’s size (Goodale et al., 1994a; Whitwell et al., 2015a). One account for this unexpected result was that when DF’s grasps were displaced next to the visible target, her fingertips inadvertently also landed on the surface of the table. This tactile influence may have been enough to recruit her functioning dorsal stream to engage in visually guided automatic grasping.

Support for this tactile feedback hypothesis came from two studies: one showing that DF retained her grip scaling to object size when she reached out to pick up 2D printed rectangular shapes as if they were 3D objects (Westwood et al., 2002); and another showing that she loses her grip scaling when she reaches out to grasp a visible virtual object that is not physically there (Schenk, 2012). This latter result also suggests that visual target information in and of itself (e.g., Castiello, 1996) is not enough to drive DF’s grip scaling. Indeed, under similar virtual circumstances, DF retains her grip scaling provided the size of the grasped object is held constant while the size of the visible target varies from trial to trial (Whitwell et al., 2014, 2015b). In fact, DF’s grip aperture partially adapts to the size of the felt target, just as normally sighted controls do, despite being unaware of the mismatch in size (Säfström and Edin, 2004; Whitwell et al., 2014, 2015b). Taken together, these studies of DF suggest that tactile contact is a critical ingredient for normal dorsal-stream grasping and that, in the absence of any end-point whatsoever, structures outside the dorsal stream (including the ventral stream) become crucially engaged, even for geometrically simple rectilinear and cylindrical shapes. It is noteworthy to point out that a similar distinction within the apraxic literature exists between pantomimed tool-use while holding the tool and pantomimed tool-use made with gestures in thin air (e.g., Buxbaum et al., 2000; Goldenberg et al., 2004; Hermsdorfer et al., 2012).

### Pantomimed Grasps as Natural Grasps Without Haptic Calibration

Perhaps the simplest account of pantomimed grasping is that it is the outcome of the natural haptics-dependent grasp system when it has been left uncalibrated by consistent absence of haptic feedback across iterative grasps. This line of reasoning is supported by the fact that many kinematic differences between grasps and pantomimed grasps vanish when haptic and non-haptic feedback trials are randomly intermixed (Bingham et al., 2007). Nevertheless, we suspect there is more to it than that, for at least three reasons. First, the haptic calibration account does not accommodate the possibility that cognitive supervision (e.g., Norman and Shallice, 1986; Shallice and Burgess, 1993) directly influences crucial parameters of pantomimed (uncalibrated) grasps such as hand aperture. Bingham et al. (2007) left open the possibility that cues to the availability of haptic feedback could shift control between pantomime and natural modes of grasping, because the expectation for haptic feedback was never manipulated independently of the trial schedule of haptic conditions. Without intermixing the two haptic conditions and manipulating expectations for haptic feedback, one cannot disentangle the influence of sensorimotor calibration from that of cognitive supervision. Second, the haptic calibration account cannot explain why pantomime grasps would be more susceptible than natural grasps to pictorial illusions (e.g., Westwood et al., 2000; Rinsma et al., 2017), particularly when the illusion is correlated with activity in the ventral stream structures, including LOC, that is early enough (< 300 ms) to putatively influence the grasp (Weidner et al., 2010). Third, haptic calibration cannot explain why the pantomime grasps performed by magicians more closely resemble “calibrated” natural grasps (Cavina-Pratesi et al., 2011; Rinsma et al., 2017), despite the absence of haptic feedback. Moreover, a magician’s expertise in pantomime grasping does not confer immunity from the illusory effects of the Muller-Lyer illusion on displaced pantomime grasps made to simulate picking up 3D objects subjected to the illusion (Rinsma et al., 2017).

We take the “pantomime” grasps of naive participants as an example of performance under direct cognitive control or cognitive supervision. For “automatic” grasps, the selection and specification of parameters like wrist orientation, reach velocity and grasp aperture size (or the paths the fingers take) occurs with minimal awareness and minimal cognitive control, as is clearly the case for DF and in cases of action blindsight, in which the person cannot reliably perceive the geometry and spatial disposition of the goal object. Cognitive supervision under these more natural circumstances maintains focus on the overarching goal of the movement (which is typically to *do something* with the object) rather than on the details of the unfolding movement in real-time. Conversely, for grasps in which parameter specification is under direct cognitive control, cognitive supervision is focused on the unfolding movement in real-time, rather than on the overarching goal. Pantomime grasps and grasps made iteratively without haptic feedback exhibit signs of cognitive control: Relative to natural grasps, they take longer to initiate, the movement is slower, the hand’s in-flight aperture is typically smaller, and the aperture’s scaling to the size of the target is more variable, i.e., some participants use their hand aperture to grossly exaggerate the differences among target sizes, whereas others hardly bother to differentiate the target sizes at all (e.g., Bingham et al., 2007; Fukui and Inui, 2013; Whitwell et al., 2015a).

### Study Overview

Here we tested the possibility that pantomime grasps performed by adults naïve to the task and directed at virtual objects are influenced by expectations about the availability of haptic feedback at the end of the reach. Note that this possibility does not negate the role of grip calibration from recent haptic end-point feedback (e.g., Bingham et al., 2007; Volcic and Domini, 2018). We fully expected that grip calibration would carry over from one trial to the next, based on the large body of work showing that, for example, grip aperture adjusts to mismatches between the haptic and visual size of the goal object, even in the absence of awareness of the mismatch (e.g., Säfström and Edin, 2004, 2008; Weigelt and Bock, 2007; Coats et al., 2008; Karok and Newport, 2010). Rather, we were asking here whether participant’s explicit haptic expectations about the grasp they are about to perform can make unique contributions to grasp parameters over and above that of the calibration. Put another way, we tested the cognitive penetribility of grasps, in so far as this cognitive effort can exploit reliable cues to the availability of haptic feedback.

Our experimental design borrows from previous work that varied expectations for the availability of *visual* feedback in order to test the cognitive penetrability of grasps (Whitwell et al., 2008). The authors did this by using verbal cues to manipulate the participant’s expectation for receiving online *visual* feedback on an upcoming reach and observing how these expectations influenced, if at all, a well-established effect of visual feedback on grasp aperture: grasps executed without online visual feedback exhibit wider in-flight grip aperture, compared to grasps executed *with* online visual feedback (e.g., Hesse and Franz, 2010; for review, see Smeets and Brenner, 1999; for the effects of partial removal of visual feedback, see Bozzacchi et al., 2018). Whitwell et al. (2008) organized the two visual feedback conditions into four different sets of trials: two sets, one for each visual condition (blocked); a third set in which the visual conditions predictably alternated from one trial to the next (alternating); and a fourth set in which they were randomly intermixed and unpredictable (randomized). Participants were verbally cued before the beginning of each trial set about the nature of the order of the visual condition. If expectations about the visual condition were cognitively exploitable, then performance in the alternating (predictable) trial set would look like performance when the visual conditions were blocked separately but would differ from performance in the randomized schedule, in which the visual condition was not predictable. If, in contrast, the parameterization of the grasp was influenced by previous grasps, rather than predictive information about the upcoming visual condition, then performance on the visual conditions would homogenize in both the alternating and randomized schedules, because the visual conditions are intermixed, but would dissociate in the blocked schedules in which stable visual conditions would afford optimal calibration. Whitwell et al. (2008) observed the latter outcome. In a follow-up experiment, a trial-by-trial analysis of the different visual conditions showed the performance diverges with successive trials of one visual condition compared to the other, in-line with a sensorimotor-calibration based account (Whitwell and Goodale, 2009).

We based our current experiment primarily on the design of Whitwell et al. (2008), but rather than varying the expectations for *visual* feedback, we vary the expectations for *haptic* feedback. We did this by comparing grasps made with and without haptic feedback into five different sets of trials: in two sets of trials, the two haptic conditions were blocked separately (*blocked*); in a third set of trials the haptic conditions alternated predictably from one trial to the next (*alternating*); and in a fourth and fifth set of trials, the haptic conditions were randomly intermixed with identical trial orders (*randomized*). Crucially, in one of these two randomized schedules, the availability of haptic feedback was made known to the participant in advance (*random cued* vs. *random uncued*). The two randomized schedules possess the same sequence of haptic conditions and are, therefore, controlled for trial-to-trial haptic-calibration based influences.

This design affords two approaches to data analysis: First, we performed targeted tests for the influence of the cues on block-level performance. This analysis addresses the hypothesis that reliable expectations regarding haptic feedback can promote pantomime-like grasps on upcoming trials *without* haptic feedback and more natural-like grasps on upcoming trials *with* haptic feedback. Thus, support for the role of cues and cognitive control should be reflected in a larger effect of removing haptic feedback in the randomized trial set with cues than in the one without cues. Second, we tested the unique influences of (1) the pending haptic condition on the current trial (t) on grip aperture and (2) the haptic condition on the immediately preceding trial (*t* − 1). If cues about the availability of haptic feedback can flexibly switch the response-mode between natural and pantomime grasps, as cognitive control (cognitive penetrability) would predict, then we should observe an influence of the pending, cued haptic condition on grip aperture and little or no influence of the immediately preceding haptic condition. Furthermore, in the absence of cues, we should observe a greater influence of the immediately preceding haptic condition on grip aperture and no influence of the pending and unknown (to the participant) haptic condition on grip aperture.

## Materials and Methods

### Participants

Thirty right-handed participants with normal or corrected-to-normal vision were recruited at the University of Western Ontario (20 females, aged 18–57 years (*M* = 22.4, *SD* = 7.99). Participants provided written and informed consent prior to participating in the study. Ethical procedures were approved by the local ethics committee. Participants were compensated $10 for their time, and they were naïve to the purpose of the study.

### Apparatus and Experimental Setup

**FIGURE 1:**
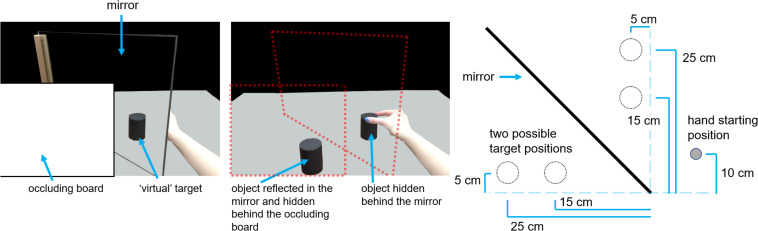
Mirror apparatus and setup. Illustration of the participant’s view of the virtual target (leftmost panel), the occluding board and mirror removed to reveal the object reflected in the mirror, and the identically sized object hidden behind the mirror and positioned to as to match the apparent position of the virtual object (middle panel), and a schematic bird’s-eye view of the setup, including the mirror, the hand’s approximate starting position, and the two possible object locations, which are indicated with circles with dotted outlines (rightmost panel).

Figure 1 shows the experimental setup, which employed a mirror-apparatus allowing the experimenter to manipulate the presence or absence of haptic feedback (e.g., Whitwell et al., 2015a). In brief, the mirror-apparatus comprised an upright mirror with reflective side perpendicular to the horizontal plane and oriented 45° clockwise from the edge of the table nearest the participant. When seated, the participants faced the reflective side of the mirror at the 45° clockwise orientation.

The participant’s starting location was a microswitch that was fixed to the table surface 10 cm away from the table edge along the participant’s sagittal plane. Between trials, and before the start of each reach, the participant used their index finger and thumb, pinched together, to depress the microswitch. The microswitch was used to help control the state of the lenses of a pair of PLATO goggles (Translucent Technologies, Toronto, ON, Canada). The lenses of the goggles can switch between the two states (translucent and transparent) in < 6 ms. The lenses of the goggles assumed a default translucent state that blocks the wearer’s view. The lenses remained translucent between trials but were cleared at the start of each trial to reveal the workspace, which included the virtual target cylinder. When participants released the microswitch at movement initiation, the lenses switched back to their default translucent state. Thus, participants did not receive visual input about the target or their hand throughout their reach; actions performed under these circumstances are referred to as being performed in visual “open loop.”

The principal reason for using open loop stemmed from the fact that the mirror obstructs the view of the participant’s hand throughout most of the reach. Using an open loop procedure in this context means that the participant does not have the experience of seeing their hand disappear behind the mirror and, for the subset of movements in which their hand makes contact with an object, the experience of lifting an object behind the mirror while it remains visibly static in the mirror. In other words, the visual open loop condition was believed to minimize a reduction in immersion.

The set of target objects were three pairs of wooden cylinders painted matte black. Each pair differed only in diameter (3.5, 4.8, and 6 cm). On any given trial, any one of the three different objects was positioned in front of the mirror, so that the participant would see its reflection in the mirror (the virtual object), while its partner (an identical cylinder) could be positioned behind the mirror such that this hidden cylinder was spatially coincident with the virtual cylinder. Thus, the cylinders behind the mirror were located mirror-opposite of the cylinders in front. The cylinders in front of the mirror could be located at any one of two possible locations and the distance between them was 10 cm. To ensure a consistent and accurate placement, the cylinders each had holes drilled into the bottom, so that each cylinder could be placed onto the peg of small square wooden plates, painted matte white, that were fixed to the table surface. This was done for the cylinder locations behind the mirror as well. The distance between the start position and the closest cylinder location was ∼17 cm. The start position was allowed to vary by small amounts to suit each participant’s preference for comfortability provided it did not block the participants ability to see their hand, because (1) target distance was not a variable of theoretical interest and (2) the manipulations of theoretical interest were within-subjects.

The positions of three infrared emitting diodes (IREDs) affixed to the inner nails of the index finger and thumb, and the wrist of the right hand, was recorded with submillimeter precision at sampling rate of 200 Hz by an active optoelectronic motion capturing system, OPTOTRAK^TM^ 3020 (Northern Digital, Waterloo, ON, Canada) with a positional measurement error of < ± 21 mm.

### Procedure

The subject was seated comfortably at the table in front of the mirror and oriented so that (1) the occluding board blocked a direct view of the cylinder at each one of the two locations, but (2) did not block a binocular view of the virtual cylinder at each location. The experimenter explained the task, using demonstrations, after which the three IREDs were attached with medical tape to the participant’s right hand: one at the corner of the proximal base of the thumb nail, one at the corner of the proximal base of the index finger nail, and one at the proximal end of the index finger on the dorsal face. The medical tape was used to secure the IREDs and to ensure that the pads of the index finger and thumb were uncovered and would receive typical tactile feedback from touching objects. Note that the distance between the IREDs was non-zero and, therefore, the computed distance between the IREDs will include this additional amount.

Participants started each trial with the tips of their index finger pinched together and depressing the microswitch. They were instructed to use visibility of the workspace as the imperative to locate the virtual cylinder and then reach out behind the mirror to grasp it. Furthermore, the participants were asked to reach out naturally, neither speeded nor labored. On trials in which a cylinder was positioned behind the mirror (haptic feedback trials), participants were to grasp the cylinder, lift it, and move it to the right side of the table, and then return to the starting position (the microswitch). The participants were also asked to simulate grasping, lifting, and moving the virtual cylinder on trials in which no cylinder was positioned behind the mirror (no-haptic feedback trials), before returning to the starting position. The participants were asked to simulate holding the object so that the fingers would not go through it (e.g., Whitwell et al., 2015a).

Trials were organized into separate sets that differed with respect to their schedule of haptic and no-haptic feedback conditions (see section “Experimental Design”). On all trials, the experimenter positioned an object first in front of the mirror and then behind the mirror. If that trial called for no-haptic feedback, after positioning the object behind the mirror, the experimenter removed it as they withdrew their hand. This was done so that the same sequence of experimenter events and timing occurred for both the haptic and no-haptic feedback trials. Before the trial was initiated and depending on the trial set, the experimenter verbally cued the participant about whether an object was behind the mirror or not.

Between each set, the participant was invited to remove the goggles and to rest for up to 5 min. During this time, the experimenter familiarized the participant with the nature of the haptic- and no-haptic trial expectancies for the next set of trials.

### Experimental Design

Each participant was tested across five sets trial schedules. The five trial sets were dubbed *blocked* (no-haptic feedback and haptic feedback trials administered in separate blocks of trials); *alternating* haptic and no-haptic feedback; randomized haptic- and no-haptic feedback without reliable cues (*randomized uncued*); and randomized haptic- and no-haptic feedback with cues (*randomized cued*).

In a given set of trials, each cylinder was presented three times at each of the two locations in pseudorandom order for a total of 18 trials for each of the haptic and no-haptic feedback conditions (if present). Target position was manipulated to reduce the contribution of memory to the responses and to keep the task more engaging for the participants. Thus, the blocked haptic and no-haptic trial sets each consisted of 18 trials, and the alternating and randomized haptic- and no-haptic feedback trial-sets each consisted of 36 trials (18 for the haptic feedback condition and 18 for the no-haptic feedback condition). In total, each participant was administered 144 trials. The order in which the five trial sets were administered was pseudo-randomized from one participant to the next and counterbalanced across all participants. Notably, the presence or absence of an object hidden behind the mirror was always discovered by the participant at the end of their reach, regardless of presence or absence of the verbal cue (or what the participant believed would be the case).

### Data Processing

Positional data was Butterworth lowpass filtered at 20 Hz after which the 3D derivatives corresponding to speed and acceleration were computed for each sample frame for each IRED. Grip aperture was computed at each sample frame as the 3D distance between the IREDs positioned on the index-finger and thumb and the first derivative of this variable was computed (grip aperture velocity). Our principal dependent measure was PGA, which was isolated on each trial by operationally defining the forward reach component of the response. The first sample frame in which the velocity of the forward reach exceeded 5 cm/s for 100 ms was defined as the point at which the forward reach was initiated, and the time from trial start to the initiation of the forward reach was operationally defined as the movement preparation time (MPT). The forward reach was operationally terminated on the first sample frame in which the velocity of the wrist IRED fell below 10 cm/s. The length, in ms, of this time window comprised the movement time (MT). PGA was the largest grip aperture observed throughout the forward reach. Peak hand velocity (PHV) was the fastest velocity achieved by the wrist IRED throughout the forward reach time. In order to define the final grip aperture (FGA) for grasps made with haptic feedback and the pantomimed ones made without haptic feedback, we used the grip aperture velocity and defined FGA as the first sample frame in which the grip aperture velocity remained within a range ±1 cm/s following the sample frame in which PGA was achieved. We performed linear regression analysis to derive the slope relating target size to PGA and to FGA. Finally, the time it took from movement start to achieve PGA (tPGA) was also computed. Each trial was visually inspected to ensure the algorithm selected sensible sample frames.

### Statistical Analysis

For a given participant, the dependent measures from each trial were grouped by their corresponding unique conditions, which were the unique combinations of target size, target distance, haptic feedback, and haptic trial schedule. The means of these groupings served as the basic unit of analysis for each participant for repeated measures ANOVA (rmANOVA). The factors of interest for the rmANOVA were *haptic feedback* (available or not available) and the *schedule* in which these two conditions were administered (four variants: blocked, alternating, randomized cued, and randomized uncued). An expanded ANOVA that included target size as an additional factor was of no theoretical interest for the dependent measures other than PGA and FGA. Furthermore, for those measures the slopes relating target size to PGA and to FGA, by definition, capture the linear influence of target size on PGA. Type I error rate (alpha) was defined as 0.05 for each rmANOVA effect. Violations of sphericity were addressed using Greenhouse-Geisser adjustments to the degrees of freedom. Note that, as mentioned in section “Apparatus and Experimental Setup,” target distance was of no theoretical interest, and so the means for target size, target distance, haptic feedback, and haptic trial schedule were weighted accordingly.

Significant main effects of haptic trial schedule (or the superseding haptic feedback × schedule interaction) were analyzed using sets of orthogonal paired-samples *t*-tests. Relative to pair-wise alternatives, orthogonal tests reduce the number of comparisons made, which reduces the correction for multiple comparisons and increase statistical power, and they analyze unique, rather than redundant, variance. For the interactions, the first orthogonal contrast involved the haptic trial schedules that were most similar: the alternating trials and the randomly interleaved ones with reliable haptic cues. These condition pairs were tested, and, if non-significant, were averaged together and contrasted against either the blocked or randomized haptic condition schedule without reliable haptic cues. A non-significant difference at this stage led us to average together the three tested conditions into one to test the remaining haptic trial schedule. The alpha error rate was defined per family of tests, and the Holms step-down variant of the Bonferonni correction was used to control it at 0.05 (Holm, 1979).

Given our interest in PGA and the influence that preceding haptic availability exerts on it, we (1) performed a single paired-samples *t*-test to contrast mean PGA on and the mean slopes from the two randomly interleaved variants for a targeted check on the influence of reliable haptic cues on PGA and the slopes; and (2) we applied multiple linear regression to predict the average PGA on a given trial as a function of the haptic feedback condition of that trial and of the immediately preceding trial. We included the size and distance of the target on the given trial as covariates. For each participant, this regression was run separately for each of the two randomly interleaved haptic schedules. Four means were computed using the regression coefficients relating the availability of haptic feedback on the current trial to PGA and the availability of haptic feedback on the immediately preceding trial to PGA for the two randomly interleaved schedules. If inducing reliable expectations about the availability of haptic feedback can flexibly switch the response mode between natural and pantomime variants, then we should observe an influence of the haptic condition on the current trial and little influence of the haptic condition on the previous trial. Furthermore, in the absence of reliable haptic expectations, we should observe a larger influence of haptic feedback on the previous trial and no influence of haptic feedback on the current trial.

## Results

### General Remarks

The results of the rmANOVAs for each measure are presented in Table 1. Table 2 reflects the conditions means for several relevant dependent measures as a function of distance and haptic feedback.

**TABLE 1:** The effects tested with repeated measures ANOVA across the dependent measures.

Dependent measures		Effects								
		Schedule			Haptic feedback			Interaction		
		*F*	*p*	η*_*p*_*^2^	*F*	*p*	η*_*p*_*^2^	*F*	*p*	η*_*p*_*^2^
Grip	PGA	15.71	<0.001	0.35	6.54	<0.02	0.18	3.85	<0.03	0.12
	Slope (PGA)	9.78	<0.001	0.25	26.49	<0.001	0.48	19.31	<0.001	0.4
	FGA	5.18	<0.008	0.15	40.87	<0.001	0.59	5.55	<0.006	0.16
	Slope (FGA)	7.62	<0.001	0.21	4.95	<0.04	0.15	5.05	<0.004	0.15
Transport and temporal	MPT	3.99	<0.02	0.12	44.28	<0.001	0.6	21.99	<0.001	0.43
	PHV	1.47	>0.23	N/A	76.2	<0.001	0.72	28.14	<0.001	0.49
	tPGA	4.14	<0.01	0.13	29.74	<0.001	0.51	28.97	<0.001	0.5
	MT	2.09	>0.1		26.03	<0.001	0.47	12.9	<0.001	0.31

**TABLE 2:** Dependent measures as a function of target distance (‘near’ and ‘far’) and haptic condition.

Dependent measures		Haptic feedback		No haptic feedback	
		Near	Far	Near	Far
Grasp	*PGA*(*mm*)	88.5 (8)	91.3 (8.8)	86.2 (10.3)	88.4 (10.9)
	*FGA*(*mm*)	62.1 (2.5)	62.1 (2.7)	55.8 (6.5)	54.8 (6.6)
Transport and temporal	*PHV*(*mm*/*s*)	477 (90)	600 (108)	444 (92)	565 (102)
	*RT*(*mm*)	655 (175)	676 (184)	714 (185)	722 (187)
	*tPGA*(*ms*)	298 (59)	332 (75)	314 (56)	344 (69)
	*MT*(*ms*)	740 (91)	872 (107)	785 (102)	909 (113)

**FIGURE 2:**
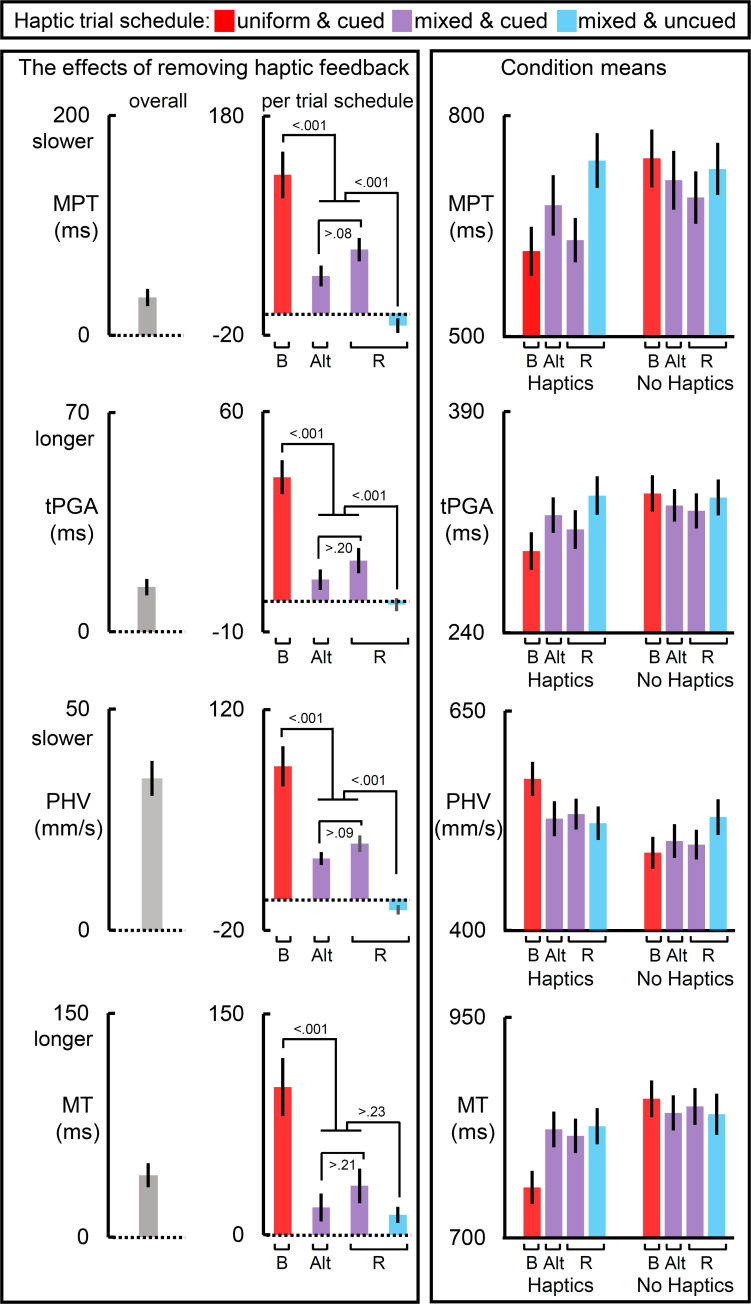
The effects of removing haptic feedback on temporal and transport measures. The leftmost and middle columns of panels reflect the effect of removing haptic feedback on movement preparation time (MPT), time to achieve peak grip aperture (tPGA), peak hand velocity (PHV), and the movement time (MT). Removing haptic feedback slowed MPT, PHV, and MT, and prolonged tPGA. Most notably, the schedule of haptic feedback modulated these effects (middle column of panels). On each of these measures, the blocked scheduled (red bars) yielded the largest effect, the cued mixed schedules yielded an intermediate one, and the uncued randomized schedule yielded the weakest effect (with the exception of MT). The raw condition means for each measure are depicted in the rightmost column of panels. Bar label “B” refers to the blocked trial order; “Alt.” alternating block of trials; and “R” randomized ones. Dotted lines reflect null of removing haptic feedback. All error bars reflect SEMs, and the dotted lines reflect the null hypothesis for tests against zero.

**FIGURE 3:**
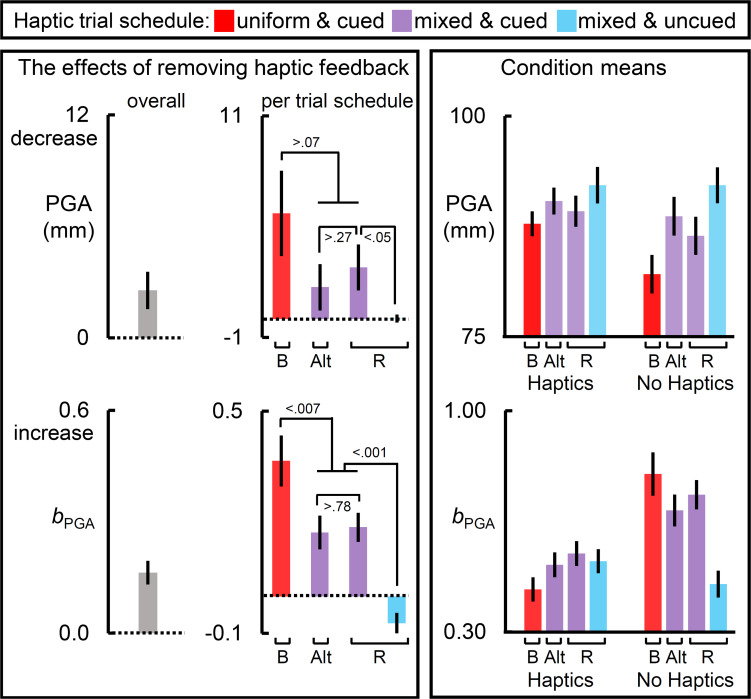
The effects of removing haptic feedback on the in-flight grip component measures. The leftmost and middle columns of panels reflect the effects of removing haptic feedback on peak grip aperture (PGA) and the slope relating target size to PGA (*b*_PGA_). Removing haptic feedback reduced PGA and increased *b*_PGA_. Most notably, the schedule of haptic feedback modulated these effects (middle column of panels). On each measure, the blocked schedule yielded the largest effect, the cued mixed schedules yielded an intermediate one, and the randomly interleaved uncued schedule yielded the weakest effect. The raw condition means for each measure are depicted in the rightmost panels. Bar label “B” refers to the blocked trial order; “Alt.” alternating block of trials; and “R” randomized ones. Dotted lines reflect a null effect of removing haptic feedback. All error bars reflect SEMs, and the dotted lines reflect the null hypothesis for tests against zero.

**FIGURE 4:**
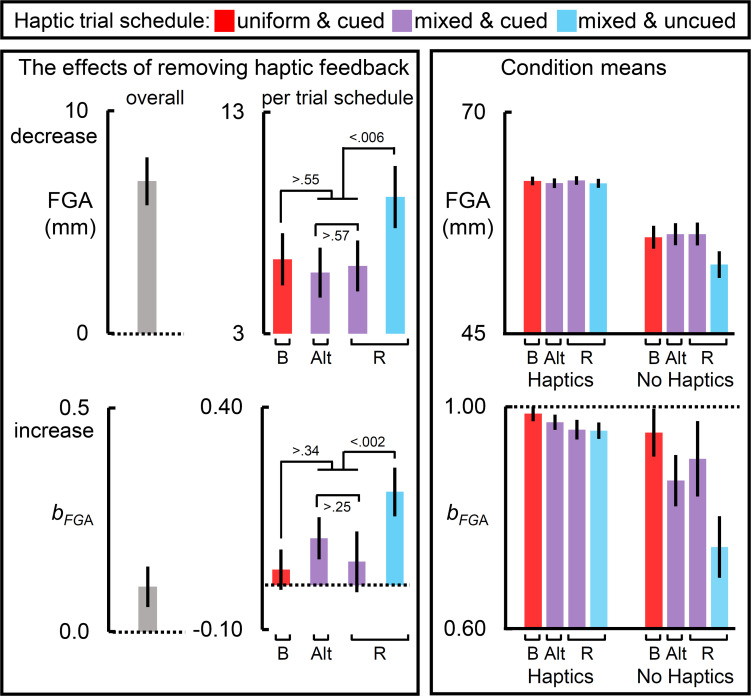
The effects of removing haptic feedback on the “final” grip component measures. The leftmost and middle columns of panels reflect the effects of removing haptic feedback on the final grip aperture (FGA) and the slope relating target size to FGA (*b*_FGA_). Removing haptic feedback decreased FGA and increased *b*_FGA_ (leftmost panels). Most notably, the schedule of haptic feedback modulated these effects (middle column of panels). On each measure, the blocked scheduled yielded the largest effect, the cued mixed schedules yielded an intermediate one, and the randomly interleaved schedule yielded the weakest effect. The raw condition means for each measure are depicted in the rightmost panels. Bar label “B” refers to the blocked trial order; “Alt.” alternating block of trials; and “R” randomized ones. Dotted lines reflect a null effect of removing haptic feedback. All error bars reflect SEMs, and the dotted lines reflect the null hypothesis for tests against zero (left panels and middle bottom panel) or one (bottom right panel).

All measures showed a main effect of removing haptic feedback, which can be viewed in the leftmost panels of Figures 2–4. All measures except PHV and MT showed a main effect of haptic schedule. All measures showed an interaction of haptic feedback and schedule, which is illustrated in the middlemost and rightmost panels of Figures 2–4. The middle panels reflect the effect of removing haptic feedback on each schedule. The rightmost panels show the condition means. In Figures 2–4, the red bars reflect the blocked schedules in which the haptic conditions were unmixed; the blue bars reflect the mixed uncued schedule; while the purple bars reflect the mixed cued schedules.

The pattern of results shown here for haptic feedback are, at first blush, strikingly similar to the results for visual feedback (c.f., Whitwell et al., 2008). For example, the largest influence of haptic feedback, like that of visual feedback in the earlier studies, occurred under the blocked schedules and the smallest differences for the mixed ones. Nevertheless, a crucial difference emerged in that there were clear differences between the cued mixed schedules, on one side, and the randomized uncued schedule on the other. These differences are expanded on below.

For each measure, we found a null difference between the alternating haptic schedule and the randomly interleaved haptic schedule with reliable cues. The only difference between these two schedules was the opportunity, in the randomly interleaved schedule, to encounter small runs of trials with or without haptic feedback. Note too that in both of these haptic trial schedules, participants were given cues concerning haptic feedback.

### Transport and Temporal Component Measures

The left panels of Figure 2 show the results for temporal measures. Removing haptic feedback increased MPT, slowed the PHV, increased the time taken tPGA and increased the MT. The middle panels of Figure 2 show that these effects were most prominent in the blocked schedules. In the mixed schedules these haptic effects were significantly reduced but not abolished entirely provided reliable haptic expectations were available. When haptic expectations were unreliable, as they were in the uncued randomized schedule, the haptic effect was null for all measures except for the MT, which was 12 ms longer without haptic feedback than with it. This likely reflects the brief continued movement of the hand when there is no object for the fingers to make contact with and the participant has no expectation for haptic feedback. The right panels of Figure 2 show that an absence of reliable expectation is similar to the effect of removing haptic feedback in terms of the direction of its effect on each measure. More generally, the right panels show that the measures on no-haptic feedback trials were generally less affected by schedules than they were on haptic feedback trials.

### In-Flight Grip Component Measures

Figure 3 shows the results for the in-flight grip component measures, PGA and the slope relating target size to PGA (*b*_PGA_).

The leftmost panels of Figure 3 show that removing haptic feedback reduced PGA and exaggerated the *b*_PGA_. The middle panels show that these haptic effects were strongest in the blocked schedule, intermediate in the cued mixed schedules, and, in the uncued randomized schedule, negligible for the PGA but, interestingly, reversed for the *b*_PGA_. For PGA, the haptic effect was greater in the blocked schedule than in the randomized uncued schedule [*t*(29) = 2.41, *p* < 0.03], and significantly greater in the randomized cued schedule than in the randomized uncued schedule [*t*(19) = 2.06, *p* < 0.05]. For PGA, the right panel shows that the mixed schedules increase PGA in both haptic conditions, but more so in the no haptic feedback condition. Furthermore, the PGA was greatest in the uncued randomized schedule. For *b*_PGA_, the right panel shows that, relative to the blocked schedules, the mixed cued schedules reduced *b*_PGA_ on no-haptic feedback trials but increased *b*_PGA_ on haptic feedback trials. This pattern was the most extreme in the uncued randomized schedule in which haptic effect reversed.

### “Final” Grip Component Measures

Figure 4 shows the results for the “final” grip component measures, FGA and the slope relating target size to FGA (*b*_FGA_).

The leftmost panels of Figure 4 show that removing haptic feedback reduced the mean FGA and exaggerated the mean *b*_FGA_. The middle panels show that, for the FGA, the haptic effect was equivalent across the schedules in which cues were available. The haptic effect on FGA was greatest in the uncued random schedule. As the rightmost FGA panel shows, this was primarily driven by the much smaller mean FGAs observed when haptic feedback was not available in this schedule. The middle panels also show that, for *b*_FGA_, the haptic effect was weakest in the cued schedules, and greatest in the uncued randomized schedule. The rightmost panel for the *b*_FGA_ show that mean *b*_FGA_ approached unitary in all of the haptic feedback conditions, regardless of schedule, which would be expected given that participants were most likely to be holding the target at that point. The haptic effect on *b*_FGA_ was greatest in the uncued randomized schedule. This was driven by the no-haptic feedback condition in which participants exhibited a much smaller mean slope.

### Peak Grip Aperture as a Function of Pending and Immediately Preceding Haptic Feedback

As described in section “Materials and Methods,” we used multiple regression to predict, for each participant, PGA as a function of the current haptic feedback condition and the immediately preceding one for the cued and uncued versions of the randomized schedules. If cues to haptic feedback are used predictively, then the main haptic contribution to PGA should stem from reliable expectation for haptic feedback on the current trial, rather than on the availability of haptic feedback on the immediately preceding trial. In the absence of reliable cues, the biggest haptic contribution to PGA should stem from the availability of haptic feedback on the immediately preceding trial.

**FIGURE 5:**
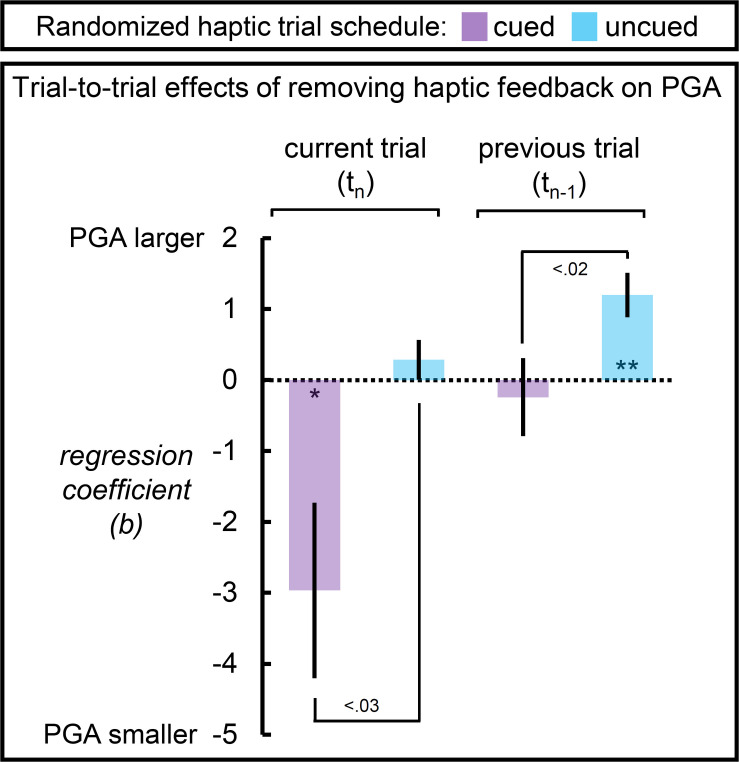
Peak grip aperture as a function of the unique effects of removing haptic feedback on the current (upcoming) and immediately preceding trial for the randomized haptic trial schedules. When participants were reliably cued that haptic feedback would not be available on the current trial, PGA decreased; but when cues were unreliable, the availability of haptic feedback on the current trial was not predictive of PGA on the current trial. The reverse pattern was observed with respect to the availability of haptic feedback on the previous trial. When cues were reliable, the availability of haptic feedback on the previous trial was not predictive of PGA; but when cues were unreliable, an absence of haptic feedback on the previous trial led to an increase in PGA. Dotted line reflects a null effect of the haptic feedback removing haptic feedback. Error bars reflect SEMs. *p < 0.05; **p < 0.01.

Figure 5 shows that this pattern is exactly what we found. On a trial-to-trial basis, participants took advantage of reliable cues regarding haptic feedback to switch between two modes of responding: larger PGA in accurate anticipation of grasping an object and smaller PGA in accurate anticipation of grasping thin-air. Furthermore, in the absence of reliable cues, we find that the haptic feedback condition on the immediately preceding trial but not the pending one, influenced PGA. Specifically, PGA was larger following a trial in which no object was grasped (and vice versa following a trial in which an object was grasped).

## Discussion

This study examined the influences of both trial-to-trial calibration and of expectations about the availability of haptic feedback on reaching to grasp virtual objects. From the outset, we expected that the sensorimotor system responsible for natural goal-directed grasps would exploit the haptic experience on previous grasps to reduce error on subsequent grasps (e.g., Säfström and Edin, 2008). We also expected iterative grasps performed without haptic feedback to take on the stereotypical characteristics of pantomimed grasps, including slower reach velocities, longer movement planning times, smaller PGA, and an exaggerated scaling of in-flight grip aperture to target size (grip scaling). Our primary question was whether grasps under these conditions are influenced only by the trial history of haptic feedback, chiefly from the immediately preceding trial, or whether they are also influenced by cued expectations about the availability of the target for grasping. We asked this question to help us understand whether the specification of parameters for real-time grasps are cognitively accessible (and subject to cognitive supervision) or if they are refractory to them, as is thought to typically occur for natural grasps.

We implemented a task in which participants reached behind a mirror to pick up visible “virtual” objects, which were sometimes available for the hand to make contact with (haptic feedback), and at other times were not (no haptic feedback). In all haptic trial schedules except one, we provided verbal cues to the availability of haptic feedback. We reasoned that cognitively accessible operations should be capable of predictively exploiting cues about the availability of haptic feedback at the end of the reach, particularly in otherwise randomized haptic-feedback schedules. Our results demonstrate that cognitive supervisory processes can exploit verbal cues about the availability of haptic feedback to plan grip aperture for future events.

We manipulated expectations about the availability of haptic feedback by varying the availability of verbal cues to the participant before the start of the trial. In the blocked trial schedule in which an object was always available for the hand to make contact with at the end of the reach, participants’ grasps were free to be influenced both by the recent history of haptic-experience (calibration) and the expectation that the hand would make contact with the object at the end of the reach. In the blocked trial schedule in which an object was not present behind the mirror and so the hand grasped “thin-air,” the expectation for no haptic feedback was available but no haptic information was available to calibrate subsequent grasps. The blocked schedules led to the greatest differences in haptic effect across all measures tested, in line with previous work drawing similar comparisons (e.g., Bingham et al., 2007; Whitwell et al., 2015a).

In three additional haptic condition schedules, the object was available on some trials and not on others, permitting intermittent opportunities for haptic calibration to occur. One set consisted of a strict schedule of alternation between haptic feedback and no feedback, allowing participant expectations about feedback to potentially be based on the regular pattern and/or their awareness of the alternating schedule. The two remaining sets consisted of randomized haptic schedules. Crucially, a verbal cue about the availability of haptic feedback on the upcoming trial was provided in only one of them. If grasps are dominated by dorsal stream operations that are inaccessible to cognitive supervisory processes, then the grasps should not dissociate as a function of the reliability of haptic expectations (as indexed by the reliability of the cues). Alternatively, if an absence of haptic feedback induces a switch to a pantomime mode of responding, recruiting structures outside the dorsal stream that are accessible to cognitive supervisory processing, then reliable haptic expectations in the cued randomized schedule should dissociate grasps made with and without haptic feedback, such that the former should look more like natural grasps while the latter should look more like pantomimed ones. Our findings support the alternative proposal. The haptic effects in the mixed schedules were reduced, relative to the effects observed in the blocked schedules, in-line with the influence of intermittent haptic calibration. Crucially, however, for all measures except for MT, reliable haptic expectations led to larger haptic effects relative to when haptic expectations were unreliable, indicating that cognitive supervisory processing can flexibly intervene to assume limited control over the response.

The analysis of PGA as a function of haptic feedback on the upcoming and immediately preceding trials in the cued and uncued randomized schedule further support the alternative viewpoint. First, with reliable haptic expectations on the upcoming trial, PGA was larger if haptic feedback was available on the upcoming trial and smaller when it was not, in-line with what one would predict if cognitive supervisory processing was flexibly engaged. Furthermore, the haptic feedback condition on the immediately preceding trial was discounted, making no contribution to PGA. In other words, reliable cues rendered grip aperture predictive with respect to the upcoming haptic condition. Second, in the absence of reliable haptic expectations, PGA was influenced by the haptic feedback condition on the immediately preceding trial *but not* the upcoming haptic condition. Specifically, PGA was smaller if haptic feedback was experienced on the immediately preceding trial than if it was not. There are two explanations for this second result. One interpretation is that without reliable cues, participants attempt to predict whether or not their hand would encounter an object on the upcoming trial, anticipating the opposite of their experience on the previous trial. Notably, this strategy would necessarily fail to differentiate the response to haptic and no-haptic feedback conditions across the trial set, because the haptic conditions are randomized. Furthermore, this explanation does not seem plausible, because participants were aware of the random nature of the haptic condition. A competing interpretation uses these considerations to suggest that participants simply adopt a wider “fail safe” margin in their PGA, resulting in the largest PGAs in this schedule (see top right panel of Figure 3); Experiencing haptic feedback on the previous trial, however, presents an opportunity to calibrate grip aperture toward the relatively smaller values more typical of natural grasps, relative to the increased PGA adopted by participants in this schedule; Whereas an absence of haptic feedback on the previous trial disrupts this process, leading to an increased safety margin on the subsequent grasp. Calibration, under the randomized uncued circumstance, competes between natural and a fail-safe modes of grasping.

The importance of both factors—calibration and cognitive control—in distinguishing pantomimed from natural grasping was also apparent in the analysis of the slopes relating target size to PGA (grip scaling). Grip scaling has been shown to be exaggerated more for pantomime grasps than for natural ones (e.g., Whitwell et al., 2015a). Our findings both reinforce and refine this observation. When cues for haptic feedback were reliable, grip scaling was greater on trials without haptic feedback than trials with it. The absence of reliable cues for haptic feedback, however, reversed this trend. In other words, when the availability of haptic feedback was unknown, grip scaling was greater on upcoming trials with haptic feedback than without it. Our explanation for this tendency is that under cognitive supervision, participants respond categorically or ordinally, rather than metrically (e.g., Dijkerman et al., 1998). This exaggerates the difference between the largest and smallest target sizes, particularly when there is no physical consequence for under or oversizing the grip aperture on no haptic feedback trials.

Our findings are consistent with those from a previous investigation that tested memory-guided pantomime grasps directed at the remembered location of a previewed object with and without haptic feedback (Davarpanah Jazi and Heath, 2016). In the haptic-feedback condition, after the participants completed their pantomime grasp, the previewed target was placed between their thumb and index finger. The haptic and no-haptic conditions were blocked separately or randomly interleaved and left uncued. The authors found that grip aperture violated Weber’s law, which has been adopted as index of absolute vs. relative coding of target size (e.g., Ganel et al., 2008; Holmes et al., 2013; Manzone et al., 2017; Ozana and Ganel, 2018b; but see Foster and Franz, 2013; Löwenkamp et al., 2015), for the haptic conditions only when blocked separately (Davarpanah Jazi and Heath, 2016). It is unknown, however, whether their findings are due to a cued expectation for haptic feedback or to visuo-haptic calibration, because the authors did not test a cued variant of their randomized haptic schedule.

Our findings are also consistent with previous work using a similar virtual setup to test the effects of visual-haptic mismatches in target size on grasping (Gentilucci et al., 1995). In one of their experiments, the authors alerted the participants to the fact that the visual and haptic sizes of the targets would not match. The authors observed a wider grip aperture that peaked earlier in the reach (Gentilucci et al., 1995). These effects are observed when participants adopt a more cautious strategy, and so it is possible that the prospect of visual haptic mismatches encouraged a cautious strategy. Nevertheless, the findings make it clear that some aspects of natural grasps made in virtual environments are amenable to coarse cognitive control.

It will be important for future studies to examine more closely the ability of the dorsal stream to use calibration and to form expectations when it is dissociated from the ventral stream. A recent study has shown that an individual, MC, with large bilateral lesions to occipital cortex cannot only point to visual targets but is also susceptible to the effects of lateral shifts in the visual field that can be introduced with prism goggles (Striemer et al., 2019). Over the course of many trials, MC’s reaches compensate for the error introduced by the prisms, indicating that the dorsal stream is capable of supporting a remarkable degree of calibration in the absence of normal phenomenological vision. Furthermore, MC also demonstrated a typical prism induced after-effect, such that, after the prisms were removed, her compensatory reaches persisted for several trials before returning back to their baseline accuracy levels (Striemer et al., 2019).

As we reviewed in the introduction, patient DF’s ventral stream is compromised and yet, when instructed to direct her pantomimed grasps beside a nearby visible target, her grasp aperture covaried reliably with target size. How is the dorsal stream using tactile feedback in DF’s case to automatically scale her grip aperture to the size of the target? The present results point to a range of possibilities. At the simplest extreme, the dorsal stream may only be able to benefit from calibration based on the most recent experience of tactile feedback. At an intermediate level, it may be able to harness memory for simple scheduling patterns such as an alternating sequence. At the most sophisticated level, it may be able to take full advantage of learned associations between reliable cues and their predictive relations to haptic feedback. We speculate, based on the literature we have reviewed, and of the role played by expectations in the present study, that the dorsal stream is only capable of calibration. But this question deserves a closer look in patients without functioning ventral streams and/or in studies using transcranial magnetic stimulation to temporarily disrupt the ventral stream contribution to a grasping task.

These findings also have important practical implications. One ready application is in virtual reality environments, where the aim is to enhance the user’s immersive experience by simulating manual interactions with virtual objects as realistically as possible. Our findings reinforce a broad observation that participants tend to adopt pantomime styled grasps in these settings that consist of stereotypically different kinematic characteristics than do natural grasps. When combined with the virtual environment, motion tracking equipment sensitive enough to resolve fine movements of the fingers that occur during reach to grasp actions may benefit from algorithms that interpret the resultant kinematic data in a way that matches the agent’s actual movements to virtual ones that more closely resemble natural grasps. Recent work has shown that although naïve individuals are poor at discriminating videos of pantomimed and natural grasps, magicians perform at above-chance levels, suggesting that expertise in sleight of hand and pantomime enhances discrimination of fine-grained differences between pantomimed and natural grasps (Quarona et al., 2020). Although it is clear from our experience that many individuals find grasping thin-air unusual, it is not clear whether naïve participants can use kinesthetic feedback to discriminate pantomimed and natural grasps. Mapping pantomime grasps to stereotypical, natural-looking grasps in visual virtual space may be a valid approach if users are unlikely to detect differences between their kinesthetic and visual experience; by that same token, a spatiotemporal mapping that closely approximates a point-to-point relationship of pantomimes to virtual movements may be necessary if users are likely to detect the discrepancy and experience a concomitant reduction in immersion^1^.

Future work should examine the computational basis for inferring an agent’s intended goal when they perform pantomime reaching and grasping in more realistic virtual environments with cluttered spaces and multiple possible goal objects within reach. Existing research has shown that an individual’s intentions for a given grasp (e.g., to pour vs. to drink from a glass) are available in the visible kinematics (Koul et al., 2019). Studies that combine these techniques with measurements of the agent’s level of immersion could track the effect that a closer mapping between intention, action, and environmental consequence has on immersion.

## Data Availability Statement

The raw data supporting the conclusions of this article will be made available by the authors, without undue reservation, to any qualified researcher.

## Ethics Statement

The studies involving human participants were reviewed and approved by the Behavioural Research Ethics Board Western University. The patients/participants provided their written informed consent to participate in this study.

## Author Contributions

RW designed the experiment. NK administered the experiment. RW and NK analyzed the data. All authors contributed to writing of the manuscript.

## Conflict of Interest

The authors declare that the research was conducted in the absence of any commercial or financial relationships that could be construed as a potential conflict of interest.

## Footnotes

1 Here, “immersion,” refers to the extent to which the VR experience feels real. McMahan’s (2003) notion of “realism,” for example, can be extended to action execution. An agent immersed in their VR experience would seem to forget, temporarily, that they were in a VR environment. This term can apply to the entire experience or just aspects of it, like reaching out to pick up a virtual object. The agent’s knowledge that they are in a VR environment should not necessarily track the feeling that when they reach out to pick up a virtual environment, they are doing so naturally, automatically, with little cognitive effort. The agent is immersed with respect to their action.

## References

[B1] Bingham G. Coats R. Mon-Williams M. (2007). Natural prehension in trials without haptic feedback but only when calibration is allowed. *Neuropsychologia* 45 288–294. 10.1016/j.neuropsychologia.2006.07.011 17045314

[B2] Bozzacchi C. Brenner E. Smeets J. B. Volcic R. Domini F. (2018). How removing visual information affects grasping movements. *Exp. Brain Res.* 236 985–995. 10.1007/s00221-018-5186-6 29399704 PMC5887006

[B3] Brandi M.-L. Wohlschlager A. Sorg C. Hermsdorfer J. (2014). The neural correlates of planning and executing actual tool use. *J. Neurosci.* 34 13183–13194. 10.1523/jneurosci.0597-14.2014 25253863 PMC6608341

[B4] Buxbaum L. J. Giovannetti T. Libon D. (2000). The role of the dynamic body schema in praxis: evidence from primary progressive apraxia. *Brain Cogn.* 44 166–191. 10.1006/brcg.2000.1227 11041988

[B5] Camponogara I. Volcic R. (2019). Grasping movements toward seen and handheld objects. *Sci. Rep.* 9:3665. 10.1038/s41598-018-38277-w 30842478 PMC6403353

[B6] Carey D. P. Harvey M. Milner A. D. (1996). Visuomotor sensitivity for shape and orientation in a patient with visual form agnosia. *Neuropsvchologia* 34 329–337. 10.1016/0028-3932(95)00169-7 9148189

[B7] Carther-Krone T. A. Senenayake S. A. Marotta J. J. (2020). The influence of the Sander parallelogram illusion and early, middle and late vision on goal-directed reaching and grasping. *Exp. Brain Res.* 238 2993–3003. 10.1007/s00221-020-05960-2 33095294

[B8] Castiello U. (1996). Grasping a fruit: selection for action. *J. Exp. Psychol.* 22 582–603. 10.1037/0096-1523.22.3.582 8666954

[B9] Cavina-Pratesi C. Kuhn G. Ietswaart M. Milner A. D. (2011). The magic grasp: motor expertise in deception. *PLoS One* 6 1–5. 10.1371/journal.pone.0016568 21347416 PMC3036651

[B10] Chen J. Snow J. C. Culham J. C. Goodale M. A. (2017). What role does “Elongation” play in “Tool-Specific” activation and connectivity in the dorsal and ventral visual streams? *Cereb. Cortex* 28 1117–1131. 10.1093/cercor/bhx017 28334063 PMC6454576

[B11] Coats R. Bingham G. P. Mon-Williams M. (2008). Calibrating grasp size and reach distance: interactions reveal integral organization of reaching-to-grasp movements. *Exp. Brain Res.* 189 211–220. 10.1007/s00221-008-1418-5 18493753

[B12] Creem S. H. Proffitt D. R. (2001). Grasping objects by their handles: a necessary interaction between cognition and action. *J. Exp. Psychol.* 27 218–228. 10.1037/0096-1523.27.1.218 11248935

[B13] Danckert J. Rossetti Y. (2005). Blindsight in action: what can the different sub-types of Blindsight tell us about the control of visually guided actions? *Neurosci. BioBehave. Rev.* 29 1035–1046. 10.1016/j.neubiorev.2005.02.001 16143169

[B14] Davarpanah Jazi S. Heath M. (2016). Pantomime-grasping: advance knowledge of haptic feedback availability supports an absolute visuo-haptic calibration. *Front. Hum. Neurosci.* 10:197. 10.3389/fnhum.2016.00197 27199718 PMC4858644

[B15] Dijkerman H. C. Milner A. D. (1998). The perception and prehension of objects oriented in the depth plane II. Dissociated orientation functions in normal subjects. *Exp. Brain Res.* 118 408–414. 10.1007/s002210050294 9497147

[B16] Eloka O. Feuerhake F. Janczyk M. Franz V. H. (2015). Garner-interference in left-handed awkward grasping. *Psychol. Res. Psychol. Forsch.* 79 579–589. 10.1007/s00426-014-0585-1 24980084

[B17] Foster R. M. Franz V. H. (2013). Inferences about time course of Weber’s Law violate statistical principles. *Vis. Res.* 78 56–60. 10.1016/j.visres.2012.11.012 23246322

[B18] Freud E. Ganel T. (2015). Visual control of action directed toward two-dimensional objects relies on holistic processing of object shape. *Psychon, Bull. Rev.* 22 1377–1382. 10.3758/s13423-015-0803-x 25665797

[B19] Frey S. H. (2007). What puts the how in where? Tool use and the divided visual streams hypothesis. *Cortex* 43 368–375. 10.1016/s0010-9452(08)70462-3 17533760

[B20] Frey S. H. (2008). Tool use, communicative gesture and cerebral asymmetries in the modern human brain. *Philos. Trans. R. Soc. Lond. B Biol. Sci.* 363 1951–1957. 10.1098/rstb.2008.0008 18292060 PMC2606701

[B21] Fukui T. Inui T. (2013). How vision affects kinematic properties of pantomimed prehension movements. *Front. Psychol.* 4:44. 10.3389/fpsyg.2013.00044 23404470 PMC3566380

[B22] Gallivan J. P. Cant J. S. Goodale M. A. Flanagan J. R. (2014). Representation of object weight in human ventral visual cortex. *Curr. Biol.* 24 1–8. 10.1016/j.cub.2014.06.046 25065755

[B23] Ganel T. Chajut E. Algom D. (2008). Visual coding for action violates fundamental psychophysical principles. *Curr. Biol.* 18 599–601. 10.1016/j.cub.2008.04.052 18644333

[B24] Ganel T. Goodale M. A. (2003). Visual control of action but not perception requires analytical processing of object shape. *Nature* 426 664–667. 10.1038/nature02156 14668865

[B25] Ganel T. Goodale M. A. (2014). Variability-based Garner interference for perceptual estimations but not for grasping. *Exp. Brain Res.* 232 1751–1758. 10.1007/s00221-014-3867-3 24534914

[B26] Gentilucci M. Daprati E. Toni I. Chieffi S. Saetti M. C. (1995). Unconscious updating of grasp motor program. *Exp. Brain Res.* 105 291–303. 7498382 10.1007/BF00240965

[B27] Goldenberg G. Hentze S. Hermsdörfer J. (2004). The effect of tactile feedback on pantomime of tool use in apraxia. *Neurology* 63 1863–1867. 10.1212/01.wnl.0000144283.38174.07 15557503

[B28] Goodale M. A. Jakobson L. S. Keillor J. M. (1994a). Differences in the visual control of pantomimed and natural grasping movements. *Neuropsychologia* 32 1159–1178. 10.1016/0028-3932(94)90100-7 7845558

[B29] Goodale M. A. Meenan J. P. Bulthoff H. H. Nicolle D. A. Murphy K. J. Racicot C. I. (1994b). Separate neural pathways for the visual analysis of object shape in Perception and prehension. *Curr. Biol.* 4 604–610. 10.1016/s0960-9822(00)00132-9 7953534

[B30] Goodale M. A. Milner A. D. Jakobson L. S. Carey D. P. (1991). A neurological dissociation between perceiving objects and grasping them. *Nature* 349 154–156. 10.1038/349154a0 1986306

[B31] Heath M. Holmes S. A. Mulla A. Binsted G. (2012). Grasping time does not influence the early adherence of aperture shaping to Weber’s law. *Front. Hum. Neurosci.* 6:332. 10.3389/fnhum.2012.00332 23267323 PMC3527824

[B32] Hermsdorfer J. Li Y. Randerath J. Goldenberg G. Johannsen L. (2012). Tool use without a tool: kinematic characteristics of pantomiming as compared to actual use and the effect of brain damage. *Exp. Brain Res.* 218 201–214. 10.1007/s00221-012-3021-z 22349499

[B33] Hermsdörfer J. Terlinden G. Mühlau M. Goldenberg G. Wohlschläger A. M. (2007). Neural representations of pantomimed and actual tool use: evidence from an event-related fMRI study. *NeuroImage* 36 109–118. 10.1016/j.neuroimage.2007.03.037 17499158

[B34] Hesse C. Franz V. H. (2010). Grasping remembered objects: exponential decay of the visual memory. *Vis. Res.* 50 2642–2650. 10.1016/j.visres.2010.07.026 20692279

[B35] Holm S. (1979). A simple sequentially rejective multiple test procedure. *Scand. J. Stat.* 6 65–70.

[B36] Holmes S. A. Lohmus J. McKinnon S. Mulla A. Heath M. (2013). Distinct visual cues mediate aperture shaping for grasping and pantomime-grasping tasks. *J. Mot. Behav.* 45 431–439. 10.1080/00222895.2013.818930 23971991

[B37] Holmes S. A. Mulla A. Binsted G. Heath M. (2011). Visually and memory-guided grasping: aperture shaping exhibits a timedependent scaling to Weber’s law. *Vis. Res.* 51 1941–1948. 10.1016/j.visres.2011.07.005 21777599

[B38] Humphreys G. Riddoch J. (2013). *A Case Study in Visual Agnosia Revisited: To See But Not To See*, 2nd Edn Hove: Psychology Press.

[B39] Isa T. (2019). Dexterous hand movements and their recovery after central nervous system injury. *Annu. Rev. Neurosci.* 42 315–335. 10.1146/annurev-neuro-070918-050436 30939102

[B40] Jakobson L. S. Archibald Y. M. Carey D. P. Goodale M. A. (1991). A kinematic analysis of reaching and grasping movements in a patient recovering from optic ataxia. *Neuropsychologia* 29 803–809. 10.1016/0028-3932(91)90073-H 1944879

[B41] James T. W. Culham J. Humphrey G. K. Milner A. D. Goodale M. A. (2003). Ventral occipital lesions impair object recognition but not object-directed grasping: an fMRI study. *Brain* 126 2463–2475. 10.1093/brain/awg248 14506065

[B42] Jeannerod M. Dicety J. Michel F. (1994). Impairment of grasping movements following a bilateral posterior parietal lesion. *Neuropsychologia* 12 369–380. 10.1016/0028-3932(94)90084-1 8047246

[B43] Jeannerod M. Jacobb P. (2005). Visual cognition: a new look at the two-visual systems model. *Neuropsychologia* 43 301–312. 10.1016/j.neuropsychologia.2004.11.016 15707914

[B44] Karnath H. O. Ruter J. Mandler A. Himmelbach M. (2009). The anatomy of object recognition – visual form agnosia caused by medial occipitotemporal stroke. *J. Neurosci.* 29 5854–5862. 10.1523/JNEUROSCI.5192-08 19420252 PMC6665227

[B45] Karok S. Newport R. (2010). The continuous updating of grasp in response to dynamic changes in object size, hand size and distractor proximity. *Neuropsychologia* 48 3891–3900. 10.1016/j.neuropsychologia.2010.10.006 20933527

[B46] Kopiske K. K. Bruno N. Hesse C. Schenk T. Franz V. H. (2016). The functional subdivision of the visual brain: is there a real illusion effect on action? A multi-lab replication study. *Cortex* 79 130–152. 10.1016/j.cortex.2016.03.020 27156056

[B47] Koul A. Soriano M. Tversky B. Becchio C. Cavallo A. (2019). The kinematics that you do not expect: integrating prior information and kinematics to understand intentions. *Cognition* 182 213–219. 10.1016/j.cognition.2018.10.006 30347321

[B48] Kravitz D. J. Saleem K. S. Baker C. I. Mishkin M. (2011). A new neural framework for visuospatial processing. *Nat. Rev. Neurosci.* 12 217–230. 10.1038/nrn3008 21415848 PMC3388718

[B49] Löhr-Limpens M. Göhringer F. Schenk T. Hesse C. (2020). Grasping and perception are both affected by irrelevant information and secondary tasks: new evidence from the Garner paradigm. *Psychol. Res.* 84 1269–1283. 10.1007/s00426-019-01151-z 30778763

[B50] Löwenkamp C. Gartner W. HJaus I. D. Franz V. H. (2015). Semantic grasping escapes Weber’s law. *Neuropsychologia* 70 235–245. 10.1016/j.neuropsychologia.2015.02.037 25731904

[B51] MacDonald S. N. Culham J. C. (2015). Do human brain areas involved in visuomotor actions show a preference for real tools over visually similar non-tools? *Neuropsychologia.* 77 35–41. 10.1016/j.neuropsychologia.2015.08.004 26253009

[B52] McMahan A. (2003). “Immersion, engagement, and presence: a method for analyzing 3-D video games,” in *The Video Game Theory Reader*, eds Wolf M. J. P. Perron B. (New York, NY: Routledge), 67–86.

[B53] Milner A. D. Goodale M. A. (2006). *The Visual Brain in Action*, 2nd Edn New York, NY: Oxford University Press.

[B54] Manzone J. Jazi S. D. Whitwell R. L. Heath M. (2017). Biomechanical constraints do not influence pantomime-grasping adherence to Weber’s law: A reply to Utz et al. (2015). *Vision Res.* 130 31–35. 10.1016/j.visres.2016.09.018 27876512

[B55] Navon G. Ganel T. (2020). Consciously monitored grasping is vulnerable to perceptual intrusions. *Conscious. Cogn.* 85:103019. 10.1016/j.concog.2020.103019 32979618

[B56] Norman D. A. Shallice T. (1986). “Attention to action,” in *Consciousness and Self-Regulation*, eds Davidson R. J. Schwartz G. E. Shapiro D. (Boston, MA: Springer), 10.1007/978-1-4757-0629-1_1

[B57] Ozana A. Ganel T. (2018a). Dissociable effects of irrelevant context on 2D and 3D grasping. *Atten. Percept. Psychophys.* 80 564–575. 10.3758/s13414-017-1443-1 29101720

[B58] Ozana A. Ganel T. (2018b). Weber’s law in 2D and 3D grasping. *Psychol. Res.* 83 977–988. 10.1007/s00426-017-0913-3 28871420

[B59] Parikh P. J. Fine J. M. Santello M. (2020). Dexterous object manipulation requires context-dependent sensorimotor cortical interactions in humans. *Cereb. Cortex* 30 3087–3101. 10.1093/cercor/bhz296 31845726 PMC7197080

[B60] Perenin M. T. Rossetti Y. (1996). Grasping without form discrimination in a hemianopic field. *Neuroreport* 7 793–797. 10.1097/00001756-199602290-00027 8733747

[B61] Przybylski L. Kroliczak G. (2017). Planning functional grasps of simple tools invokes the hand-independent praxis representation network: an fMRI study. *J. Int. Neuropsychol. Soc.* 23 108–120. 10.1017/s1355617716001120 28205496

[B62] Quarona D. Koul A. Ansuini C. Pascolini L. Cavallo A. Becchio C. (2020). A kind of magic: enhanced detection of pantomimed grasps in professional magicians. *Q. J. Exp. Psychol.* 73 1092–1100. 10.1177/1747021820918533 32238037

[B63] Rice N. J. Tunik E. Cross E. S. Grafton S. T. (2007). Online grasp control is mediated by the Contralateral hemisphere. *Brain Res.* 1175C 76–84. 10.1016/j.brainres.2007.08.009 17888413 PMC2093953

[B64] Rinsma T. van der Kamp J. Dicks M. Canal-Bruland R. (2017). Nothing magical: pantomimed grasping is controlled by the ventral system. *Exp. Brain Res.* 235 1823–1833. 10.1007/s00221-016-4868-1 28299409 PMC5435791

[B65] Rizzolatti G. Matelli M. (2003). Two different streams form the dorsal visual system: anatomy and functions. *Exp. Brain Res.* 153 146–157. 10.1007/s00221-003-1588-0 14610633

[B66] Rosenbaum D. A. Meulenbroek R. J. Vaughan J. Jansen C. (2001). Pososture-based motion planning: applications to grasping. *Psychol. Rev.* 108 709–734. 10.1037/0033-295X.108.4.709 11699114

[B67] Säfström D. Edin B. B. (2004). Task requirements influence sensory integration during grasping in humans. *Learn. Mem.* 11 356–363. 10.1101/lm.71804 15169866 PMC419739

[B68] Säfström D. Edin B. B. (2008). Prediction of object contact during grasping. *Exp. Brain Res.* 190 265–277. 10.1007/s00221-008-1469-7 18592227

[B69] Schenk T. (2012). No dissociation between perception and action in Patient DF when haptic feedback is withdrawn. *J. Neurosci.* 32 2013–2017. 10.1523/JNEUROSCI.3413-11.2012 22323715 PMC6621711

[B70] Shallice T. Burgess P. (1993). “Supervisory control of action and thought selection,” in *Attention: Selection, Awareness, and Control: A Tribute to Donald Broadbent*, eds Baddeley A. D. Weiskrantz L. (Oxford: Clarendon Press/Oxford University Press), 171–187.

[B71] Smeets J. B. Brenner E. (1999). A new view on grasping. *Mot. Control* 3 237–271. 10.1123/mcj.3.3.237 10409797

[B72] Smeets J. B. J. Kleijn E. van der Meijden M. Brenner E. (2020). Why some size illusions affect grip aperture. *Exp. Brain Res.* 238 969–979. 10.1007/s00221-020-05775-1 32185404 PMC7181449

[B73] Striemer C. L. Enns J. T. Whitwell R. L. (2019). Visuomotor adaptation in the absence of input from early visual cortex. *Cortex* 115 201–215. 10.1016/j.cortex.2019.01.022 30849551

[B74] Styrkowiec P. P. Nowik A. M. Króliczak G. (2019). The neural underpinnings of haptically guided functional grasping of tools: an fMRI study. *NeuroImage* 194 149–162. 10.1016/j.neuroimage.2019.03.043 30910723

[B75] Tunik E. Frey S. T. Grafton S. H. (2005). Virtual lesions of the anterior intraparietal area disrupt goal-dependent on-line adjustments of grasp. *Nat. Neurosci.* 8 505–511. 10.1038/nn1430 15778711 PMC10719865

[B76] Volcic R. Domini F. (2018). The endless visuomotor calibration of reach-to-grasp actions. *Sci. Rep.* 8:14803. 10.1038/s41598-018-33009-6 30287832 PMC6172279

[B77] Weidner R. Boers F. Mathiak K. Dammers J. Fink G. R. (2010). The temporal dynamics of the muller-lyer illusion. *Cereb. Cortex* 20 1586–1595. 10.1093/cercor/bhp217 19875676

[B78] Weigelt C. Bock O. (2007). Adaptation of grasping responses to distorted object size and orientation. *Exp. Brain Res.* 181 139–146. 10.1007/s00221-007-0911-6 17333005

[B79] Westwood D. A. Chapman C. D. Roy E. A. (2000). Pantomimed actions may be controlled by the ventral visual stream. *Exp. Brain Res.* 130 545–548. 10.1007/s002219900287 10717797

[B80] Westwood D. A. Danckert J. A. Servos P. Goodale M. A. (2002). Grasping two-dimensional and three-dimensional objects in visual-form agnosia. *Exp. Brain Res.* 144 262–267. 10.1007/s00221-002-1068-y 12012164

[B81] Whitwell R. L. Buckingham G. Enns J. T. Chouinard P. A. Goodale M. A. (2016). Rapid decrement in the effects of the Ponzo display dissociates action and perception. *Psychon. Bull. Rev.* 23 1157–1163. 10.3758/s13423-015-0975-4 26555756

[B82] Whitwell R. L. Ganel T. Byrne C. M. Goodale M. A. (2015a). Real-time vision, tactile cues, and visual form agnosia: removing haptic feedback from a “natural” grasping task induces pantomime-like grasps. *Front. Hum. Neurosci.* 9:216. 10.3389/fnhum.2015.00216 25999834 PMC4422037

[B83] Whitwell R. L. Milner A. D. Cavina-Pratesi C. Barat M. Goodale M. A. (2015b). Patient DF’s visual brain in action: visual feedforward control in visual form agnosia. *Vis. Res.* 110 265–276. 10.1016/j.visres.2014.08.016 25199609

[B84] Whitwell R. L. Goodale M. A. (2009). Updating the programming of a precision grip is a function of recent history of available feedback. *Exp. Brain Res.* 194 619–629. 10.1007/s00221-009-1737-1 19266190

[B85] Whitwell R. L. Goodale M. A. Merritt K. E. Enns J. T. (2018). The Sander parallelogram illusion dissociates action and perception despite control for the litany of past confounds. *Cortex* 98 163–176. 10.1016/j.cortex.2017.09.013 29100659

[B86] Whitwell R. L. Lambert L. M. Goodale M. A. (2008). Grasping future events: explicit knowledge of the availability of visual feedback fails to reliably influence prehension. *Exp. Brain Res.* 188 603–611. 10.1007/s00221-008-1395-8 18443765

[B87] Whitwell R. L. Milner A. D. Cavina-Pratesi C. Byrne C. M. Goodale M. A. (2014). DF’s visual brain in action: the role of tactile cues. *Neuropsychologia* 55 41–50. 10.1016/j.neuropsychologia.2013.11.019 24300664

[B88] Whitwell R. L. Sperandio I. Buckingham G. Chouinard P. A. Goodale M. A. (2020). Grip constancy but not perceptual size constancy survives lesions of early visual cortex. *Curr. Biol.* 30 3680.e5–3686.e5. 32735814 10.1016/j.cub.2020.07.026

[B89] Whitwell R. L. Striemer C. L. Nicolle D. A. Goodale M. A. (2011). Grasping the non-conscious: preserved grip scaling to unseen objects for immediate but not delayed grasping following a unilateral lesion to primary visual cortex. *Vis. Res.* 51 908–924. 10.1016/j.visres.2011.02.005 21324336

